# Successful skipping of abnormal pseudoexon by antisense oligonucleotides in vitro for a patient with beta-propeller protein-associated neurodegeneration

**DOI:** 10.1038/s41598-024-56704-z

**Published:** 2024-03-18

**Authors:** Mamiko Yamada, Kazuhiro Maeta, Hisato Suzuki, Ryo Kurosawa, Toshiki Takenouchi, Tomonari Awaya, Masahiko Ajiro, Atsuko Takeuchi, Hisahide Nishio, Masatoshi Hagiwara, Fuyuki Miya, Masafumi Matsuo, Kenjiro Kosaki

**Affiliations:** 1 Center for Medical Genetics, Keio University School of Medicine, Tokyo, Japan; 2 KNC Department of Nucleic Acid Drug Discovery, Faculty of Rehabilitation, Kobe Gakuin University, Kobe, Japan; 3 Department of Anatomy and Developmental Biology, Graduate School of Medicine, Kyoto University, Kyoto, Japan; 4 Department of Pediatrics, Keio University School of Medicine, Tokyo, Japan; 5 Division of Cancer RNA Research, National Cancer Center Research Institute, Tokyo, Japan; 6 Faculty of Health Sciences, Kobe Tokiwa University, Kobe, Japan; 7 Faculty of Rehabilitation, Kobe Gakuin University, Kobe, Japan; 8 Center for Anatomical Studies, Graduate School of Medicine, Kyoto University, Kyoto, Japan; 9 Department of Drug Discovery Medicine, Graduate School of Medicine, Kyoto University, Kyoto, Japan

**Keywords:** Neurodevelopmental disorders, Transcriptomics

## Abstract

Pathogenic variants in *WDR45* on chromosome Xp11 cause neurodegenerative disorder beta-propeller protein-associated neurodegeneration (BPAN). Currently, there is no effective therapy for BPAN. Here we report a 17-year-old female patient with BPAN and show that antisense oligonucleotide (ASO) was effective in vitro. The patient had developmental delay and later showed extrapyramidal signs since the age of 15 years. MRI findings showed iron deposition in the globus pallidus and substantia nigra on T2 MRI. Whole genome sequencing and RNA sequencing revealed generation of pseudoexon due to inclusion of intronic sequences triggered by an intronic variant that is remote from the exon–intron junction: *WDR45* (OMIM #300526) chrX(GRCh37):g.48935143G > C, (NM_007075.4:c.235 + 159C > G). We recapitulated the exonization of intron sequences by a mini-gene assay and further sought antisense oligonucleotide that induce pseudoexon skipping using our recently developed, a dual fluorescent splicing reporter system that encodes two fluorescent proteins, mCherry, a transfection marker designed to facilitate evaluation of exon skipping and split eGFP, a splicing reaction marker. The results showed that the 24-base ASO was the strongest inducer of pseudoexon skipping. Our data presented here have provided supportive evidence for in vivo preclinical studies.

## Introduction

Pathogenic variants in the *WDR45* (OMIM #300526) gene on chromosome Xp11 cause neurodegenerative disorder beta-propeller protein-associated neurodegeneration (BPAN)^1,2^. Most patients are heterozygous females and male patients are nonviable, with the exception of male patients who had presumably incomplete loss-of-function variants (e.g., nonsynonymous variants). Clinical course of BPAN is bi-phasic. During first phase, the patients present with non-progressive intellectual disability and motor delay. Moreover, second phase starts at adolescence and the patients develop progressive extrapyramidal symptoms including dystonia and parkinsonism. Characteristically, magnetic resonance imaging shows iron accumulation in the substantia nigra and in the globus pallidus during the second phase. Currently, there is no effective therapy for BPAN.

Various classes of pathogenic variants of *WDR45* have been reported in patients with BPAN. Several patients with splicing abnormalities have been reported. Like any other genes, most previously reported splicing defects of *WDR45* are located in the vicinity of the exon–intron junction in BPAN. Here we report an exceptional patient with BPAN who exhibited activation of pseudoexon due to inclusion of intronic sequences triggered by an intronic variant^3^ that is remote from the exon–intron junction. We further developed antisense oligonucleotides which successfully suppressed expression of pseudoexon-containing transcripts^4^. In view of the recent progress of antisense oligonucleotide-mediated exon-skipping therapy for monogenic disorders caused by splicing abnormalities^5,6^, our data presented here have provided supportive evidence for in vivo preclinical studies.

## Patient report

The patient was a 17-year-old female patient who was the first child of healthy and non-consanguineous Japanese parents with no significant family medical history. She was born at 36 weeks of gestation with intra-uterine growth retardation. Her birth weight was 2024 g (− 1.41 SD), her length was 44.0 cm (− 1.22 SD), and her OFC was 30.0 cm (− 1.64 SD). She had severe psychomotor developmental delay and no meaningful speech. She gained head control at 4 months, eye tracking at the age of 2 years, sat up without support at the age of 5 years, and pulled up on things at the age of 8 years. She used a wheelchair. She had no distinctive facial features. Extrapyramidal signs, such as rigidity, dystonia, and tremor, began to appear at the age of 15 years. These signs were aggravated at the time of awakening and when fatigued.

**Figure 1:**
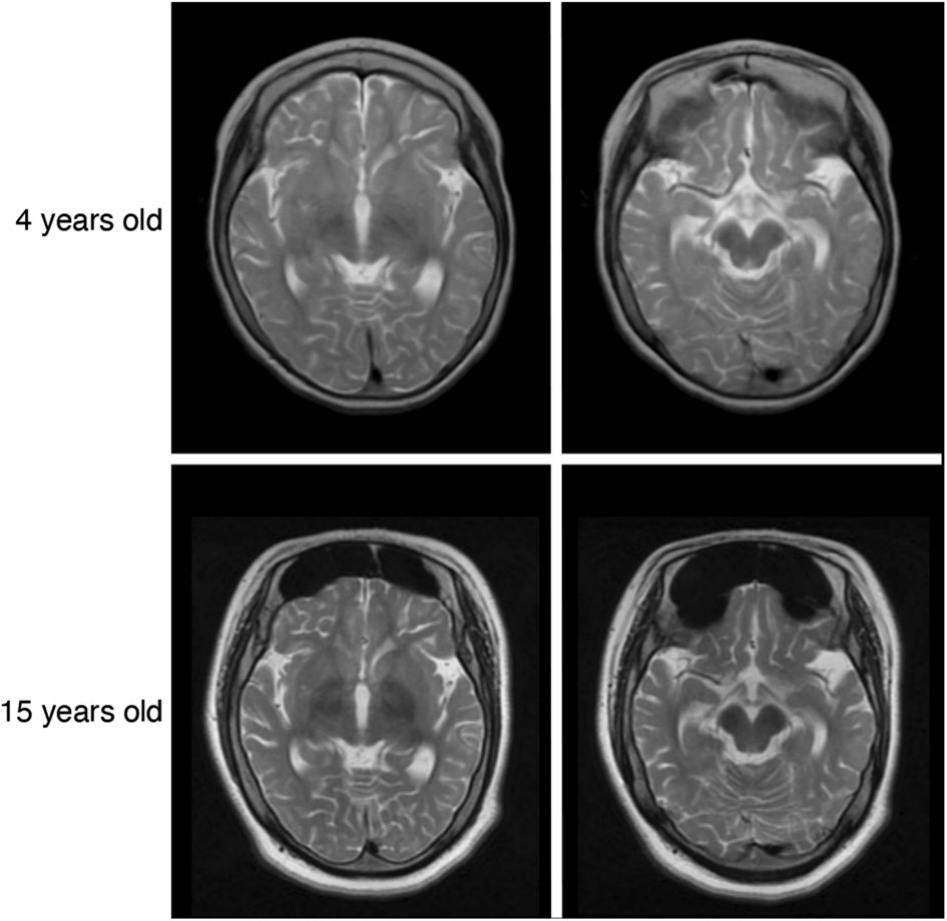
MRI at the age of 4 and 15 years. Top: MRI at the age of 4 years showed very little iron deposition in the globus pallidus (Left) and substantia nigra (Right) on T2 MRI. Bottom: MRI at the age of 15 years showed the appearance of iron deposition in the globus pallidus (Left) and substantia nigra (Right) on T2 MRI.

Magnetic resonance imaging (MRI) of the brain at the age of 4 years showed cerebral and cerebellar atrophy (Fig. 1, top left). There was very little, if any, evidence of iron accumulation in the midbrain (Fig. 1, top right). At the age of 15 years, her MRI findings had changed: iron deposition was suggested in the globus pallidus (Fig. 1, bottom left) and substantia nigra on T2 MRI (Fig. 1, bottom right). In retrospect, when the molecular diagnosis was made as below, hypointensity in T2 images was compatible with accumulation. These findings were compatible with a diagnosis of beta-propeller protein-associated neurodegeneration (BPAN).

## Results

### Exome and whole-genome analysis

Her G-banded chromosomes were normal. A trio exome analysis detected no biallelic putatively pathogenic variants and three de novo variants (*IP6K3*, *NPRL3*, and *SYN1*) within coding regions, none of which were compatible with her clinical features. Then, whole-genome sequencing and RNA sequencing were performed.

A de novo heterozygous variant was identified: *WDR45* (OMIM #300526) chrX(GRCh37):g.48935143G > C, NM_007075.4 (NM_001029896.4/ENST00000376372.9):c.235 + 159C > G. Abnormal splicing predication program SpliceAI^7^ indicated that this variant would cause gain of splicing donor site with the delta score of 0.97. The delta scores range from 0 to 1 and can be interpreted as the probability that the variant affects splicing at any position within the window around it (+ /−50 bp in the present report). PDIVAS, a recently released program, which predicts pathogenicity of deep-intronic variants was used. The variant was predicted as pathogenic with a PDIVAS score of 0.998 [0–1], well above the recommended threshold of 0.082^8^. Computer prediction programs other than SpliceAI and PDIVAS also predicted that the intronic variant would create non-canonical splicing donor site: SpliceSiteFinder, MaxEnt, NNSPLICE, and GeneSplicer gave scores of 79.74 [0–100], 8.46 [0–12], 0.79 [0–1], 2.84 [0–24], respectively. All these scores were above the threshold values for splicing donor sites [> = 70, >  = 0; >  = 0.4; and >  = 0]. Furthermore, these scores were comparable to those of the canonical splice donor site of intron 5 (100, 10.86, 1.00 and 11.04, respectively).

Heterozygous variants in *IP6K3*, *NPRL3*, and *SYN1* were detected in addition to pathogenic variants in *WDR45*. However, they are unlikely to explain the clinical features of the patient reported here. A de novo variant, NM_054111.5:c.1070C > G, p.(Pro357Arg), was identified in the *IP6K3* gene (inositol hexaphosphate kinase 3), which is not known to be associated with any human disease. The CADD score of 13.9 indicates a probable lack of clinical relevance. A de novo variant, NM_002621.2:c.1245-119_1245-118insGAA, was identified in the 5′ untranslated region of *NPRL3* (nitrogen permease regulator-like 3), which is associated with autosomal dominant epilepsy, familial focal, with variable foci 3 (OMIM:#617118). Allele frequency of 0.076 in the gnomAD database and classification as "benign" in the ClinVar database suggest a lack of clinical relevance. A de novo variant, (GRCh37)ChrX:47483957_47483958insTC, was identified in the upstream region of *SYN1* (synapsin 1) associated with an X-linked recessive neurodevelopmental/epileptic syndrome in males (OMIM:# 300491 and # 300115). A CADD score of 0.21 indicates a probable lack of clinical relevance.

### Transcriptome analysis

**Figure 2:**
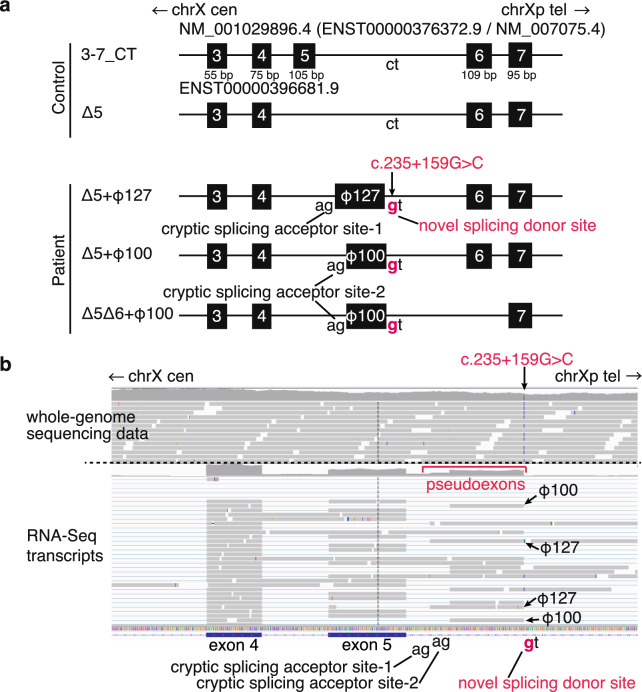
Transcriptome analysis of peripheral blood. (**a**) Schematic view of the transcriptome analysis of peripheral blood derived from the normal control (top) and from the patient (bottom). In the normal control, two types of transcripts were observed: One transcript 3-7_CT (Control) had exon 3, exon 4, exon 5, exon 6, and exon 7. The other transcript Δ5 had exons 3, the other lacked exon 3, exon 4, exon 6, and exon 7 but lacked exon 5. In the patient derived sample, three types of transcripts: The first transcript Δ5 + φ127 had exon 3, exon 4, a pseudoexon φ127, exon 6, and exon 7. The second transcript Δ5 + φ100 had exon 3, exon 4, a pseudoexon φ100, exon 6, and exon 7. The third transcript Δ5Δ6 + φ100 had exon 3, exon 4, pseudoexon φ100 and exon 7. (**b**) IGV view of the results from the whole genome analysis (top) and those from transcriptome analysis (bottom) based on genome coordinate GRCh37. The original IGV image was horizontally inverted so that transcripts are shown from left to right. Note genomic single nucleotide substitution led to creation of novel non-canonical splicing donor site and usage of cryptic splicing acceptor sites, leading to transcription of mRNA with 100 bp-long pseudoexon (φ100) or 127 bp-long pseudoexon (φ127).

Transcriptome analysis of total RNA derived from peripheral blood of the normal control showed two types of transcripts (Fig. 2a): One transcript had exon 3, exon 4, exon 5, exon 6, and exon 7 (Transcript 3-7_CT in Fig. 2a). This splicing pattern was the same as NM_001029896.4 (also known as ENST00000376372.9, the MANE transcript of *WDR45*). The other transcript had exons 3, exon 4, exon 6, and exon 7 but lacked exon 5 (Transcript Δ5 in Fig. 2a). Transcriptome analysis of total RNA derived from peripheral blood of the patient showed three types of transcripts (Fig. 2a,b): The first transcript had exon 3, exon 4, a pseudoexon (arbitrarily named φ127, Fig. 2a,b), exon 6, and exon 7 (Transcript Δ5 + φ127 in Fig. 2a). The second transcript had exon 3, exon 4, a pseudoexon shorter than φ127 (arbitrarily named φ100, Fig. 2a,b), exon 6 and exon 7 (Transcript Δ5 + φ100 in Fig. 2a). The third transcript had exon 3, exon 4, pseudoexon φ100 and exon 7 (Transcript Δ5Δ6 + φ100 in Fig. 2a). Pseudoexon inclusion of φ100 or φ127 was predicted to induce shift of the reading frame, subsequent premature termination codon (PTC), and nonsense-mediated decay: Δ5 + φ127 will lead to p.H45Ffs*77 andΔ5 + φ100 will lead to p.D44Gfs*69 at the protein level.

### Retrospective bioinformatic analysis of the activation of cryptic non-canonical splicing acceptor sites

Computer prediction programs predicted that the non-canonical splicing acceptor sites are present withing intron 5: at intron 5 − c.235 + 32 and at intron 5 − c.235 + 59. Calculated scores by SpliceSiteFinder, MaxEnt, NNSPLICE, and GeneSplicer are summarized on Supplementary Table 1. These scores were all above the threshold values for splicing acceptor sites and comparable to those of the canonical splicing acceptor site of exon 4. All these scores were above the threshold values for splicing acceptor sites.

The distribution of putative exonic splicing enhancers (ESEs, i.e., scores higher than 2.383) surrounding exon 5, φ127, φ100 and corresponding reference sequences (i.e., wild type) is shown in Supplementary Fig. 1. The intronic variant c.235 + 159G > C creates a relatively strong potential exonic splicing enhancer (SRSF2 binding site) at the 3′ end of the pseudo-exons, where SRSF2 binding sites are relatively scarce.

### In vitro proof of exonization of intron sequences

We examined whether the exonization of intron sequences shown by transcriptome analysis could be reproduced in vitro*.* We have modified the expression vector pcDNA3 to create the H492 mini-gene for splicing analysis, which has been used successfully to analyze many splicing defects^9,10^. First, insertion of exon 5 and a 988 bp fragment centered on the c.235 + 159C > G mutation, as well as a 1.45 kb region from intron 3 to intron 6 including exons 4 to 6, did not reproduce the insertion of exon 5 and a pseudoexon. It was inferred that splicing of exon 5 and the pseudoexon was placed under the control of splicing control sequences in regions further apart.

**Figure 3:**
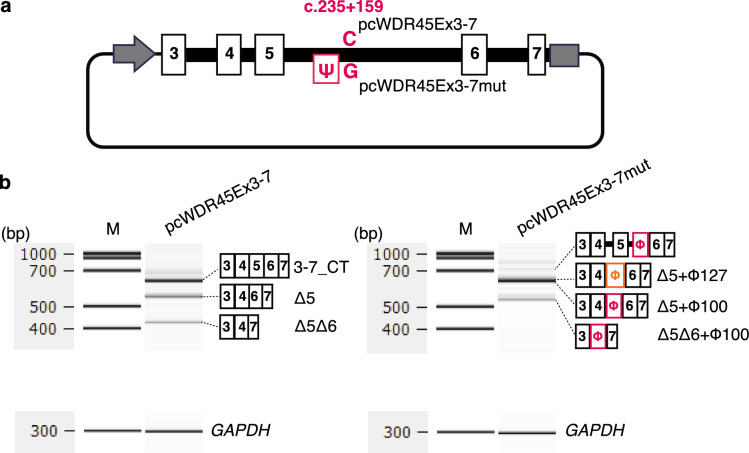
c.235 + 159C > G Splicing analysis of WDR45 minigene. (**a**) Schematic of splicing analysis mini-electrons. pcWDR45Ex3-7 (normal) or pcWDR45Ex3-7 m (patient) exons 3, 4, 5, 6, and 7 are shown in white boxes and introns 3, 4, 5, and 6 are shown in black boxes. Ψ indicates pseudoexons arisen due to the c.235 + 159C > G mutation. Gray arrow indicates a CMV promoter and gray box indicates a BGH-poly A signal. (**b**) Splicing products of the minigene. The minigene pcWDR45Ex3-7 (normal) (left) or pcWDR45Ex3-7mut (patient) (right) was introduced into HeLa cells and the mRNA was analyzed by RT-PCR. Multiple bands were detected from each (see main text), and the structure of each band is schematically noted on the right. *GAPDH* is the endogenous control. RT-PCR product from pcWDR45Ex3-7mut included pseudoexon of 100 base pairs (φ100, designated in red) or pseudoexon of 127 base pairs (φ127, designated in orange). Complete images of the gel electrophoresis were shown in Supplementary Fig. 4a and b.

Therefore, we decided to insert a 1.7 kb region of exons 3 to 7 directly into pcDNA3.1(+) to create a more naturalistic genomic structure. The homologous sequences were used to amplify from the genomic region of normal individuals and designated pcWDR45Ex3-7 (Fig. 3a). After confirming this construct by sequencing, the construct was subjected for PCR amplification using a primer set to introduce the nucleotide change. The resulting constructs were sequenced and designated pcWDR45Ex3-7mut (Fig. 3a). They were introduced into HeLa cells and the products were subjected to RT-PCR analysis.

Capillary electrophoresis of the PCR products on an Agilent 2100 Bioanalyzer (Agilent Technologies) yielded three bands as pcWDR45Ex3-7 products consisting of normal sequence. The band with the most abundance was the product including exons 3, 4, 5, 6, and 7 (3-7_CT in Fig. 3b). This pattern was the same as transcript 3-7_CT in Fig. 2b. The second most abundant band was the product including exons 3, 4, 6, and 7 but lacked exon 5. This pattern was the same as Transcript Δ5 in Fig. 2b (Δ5 in Fig. 3b). In addition, faint band was the product including exons 3, 4, and 7 but lacked exons 5 and 6 (Δ5Δ6 in Fig. 3b).

On the other hand, multiple bands were also obtained from the pcWDR45Ex3-7mut construct with the c.235 + 159C > G mutation. The amplified band with the most abundance in the product was found at approximately the same size as that in the normal, but sub-cloning sequencing revealed a mixture of two products showing pseudoexon size difference. The results showed that it was linked to exons 3 and 4, followed by a 100 bp or 127 bp pseudoexons (26 or 4 sequenced clones, respectively), and then exons 6 and 7 (Δ5 + φ100 or Δ5 + φ127 in Fig. 3b), a splicing pattern same as transcript Δ5 + φ100 and Δ5 + φ127 in Fig. 2b. The second most abundant sequence had exon 3, exon 4, φ100 and exon 7 (Δ5Δ6 + φ100 in Fig. 3b), a splicing pattern same as transcript Δ5Δ6 + φ100 in Fig. 2b. The third most abundant band was the largest in size, consisting of the full length of intron 4, exon 5, and a 158 bp sequence at the 5′ end of intron 5, followed by exon 6. All of these products inserted pseudoexons, and they were comparable to those identified in the transcriptome analysis.

### Construction of a mini-gene to search for antisense oligonucleotides that induce pseudo-exon skipping

The fact that pseudoexon insertion is the cause of BPAN indicates that inducing pseudoexon skipping during splicing would normalize the patient's gene product, and that an antisense oligonucleotide (ASO) that induces pseudoexon skipping could be a treatment for this BPAN case. Therefore, to search for ASOs that induce pseudoexon skipping, we applied our recently developed mini-gene FMv2^11^, a dual fluorescent splicing reporter system that encodes two fluorescent proteins, mCherry, a transfection marker designed to facilitate evaluation of exon skipping and split eGFP, a splicing reaction marker, that is constructed from pcDNA3.1/Hygro (+) encoding a CMV promoter, a BGH-polyA signal and a drug resistance gene (V87020, Invitrogen by Thermo-Fisher Scientific, Waltham, MA, USA). The split eGFP is mediated by an artificial intron containing a multicloning site sequence where the genomic fragment to be analyzed for splicing can be cloned into. When an exon in a genomic fragment is spliced and inserted into the transcript, the eGFP sequence is blocked and green fluorescence is not emitted. However, when exon skipping occurs, full-length eGFP is generated and green fluorescent signal is emitted. This allows FMv2 to quantify exon skipping simply by fluorescence observation using a microscope.

**Figure 4:**
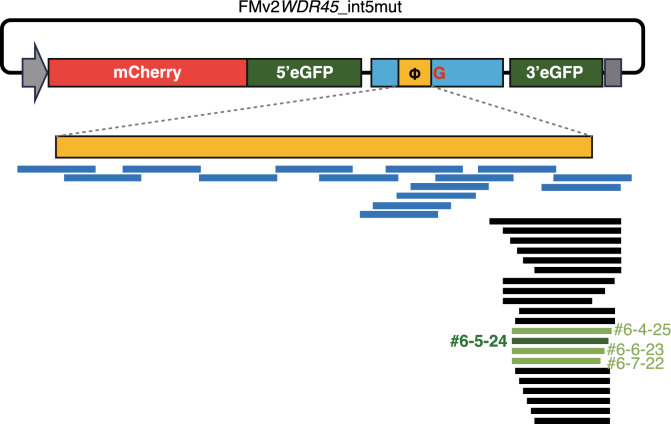
Screening strategy for antisense oligonucleotide (ASO) that would induce pseudoexon skipping. Two types of FMv2 with c.235 + 159C and G sequences were created by inserting a 641-base intron 5 sequence into the FMv2 multicloning site, and a mutated minigene was used to screen for ASO (top row). This construct with c.235 + 159C was named as FMv2WDR45_int5 (wild type). And, that with c.235 + 159G was named FMv2WDR45_mut. A total of 54 different 2′-OMe RNA-ASOs were synthesized targeting the pseudoexon region. Pseudoexon is indicated by orange lines and ASOs by blue, black, or green bars (bottom portion). ASO WDR45In5#6-5-24, the most effective ASO in the screening, is shown as a dark green bar. Other 3 relatively ASOs which were subject to further evaluation in Fig. 5. Red box indicates mCherry and green boxes indicate eGFP divided into two parts. Orange box indicates pseudoexon in the 641-base intron 5 (Blue box). Gray arrow indicates a CMV promoter and gray box indicates a BGH-polyA signal.

PCR fragments amplified from the genomic sequence of intron 5 of *WDR45* were inserted into the FMv2 multicloning site. Modified vector with wild type sequence (FMv2WDR45_int5) and modified vector with mutant sequence (FMv2WDR45_int5mut in Fig. 4) were constructed. Genomic sequences of various lengths were examined. A pseudoexon insertion was strongly observed by using a 641 bp insertion of intron 5 (Fig. 4, top) containing the mutation (Supplementary Fig. 2a bottom) whereas pseudoexon was skipped by using the same 641 bp insertion of intron 5 containing the wild type sequence (Supplementary Fig. 2 top).

### Screening for antisense oligonucleotides

Using the double fluorescence-based splicing reporter system of FMv2WDR45_int5mut described above, we screened for ASOs that promote WDR45 pseudoexon skipping. As the initial screening, we synthesized a total of 15 ASOs containing 18 mer 2′-O-methyl RNAs that covers the entire length of the 5′ to 3′ ends of the pseudoexon sequence (blue bars in Fig. 4, Table 1). They were then evaluated in terms of their activity to inhibit pseudoexon incorporation (i.e., skipping of pseudoexons to restore normal gene function).

**Table 1:** 2′-OMe-ASOs for 1st screening.

ASO name	Sequence
WDR45In5#1;	ggggugccugugggaggc
WDR45In5#2;	uucaucugccaguaccuu
WDR45In5#3;	cugaaguacucugacguc
WDR45In5#4;	gcuguaggccuccucucu
WDR45In5#5;	cuauaagauccaugacuu
WDR45In5#6;	agcaccaaccuucucaag
WDR45In5#7;	ccgagaaaucugggccaa
WDR45In5#8;	aggccaggaauccgagaa
WDR45In5#4-15;	ucugugugacucugaagu
WDR45In5#4-6;	ggccuccucucuguguga
WDR45In5#4-3;	guaggccuccucucugug
WDR45In5#4 + 3;	caagcuguaggccuccuc
WDR45In5#4 + 6;	uuccaagcuguaggccuc
WDR45In5#4 + 12;	caugacuuccaagcugua
WDR45In5#6-3;	accaaccuucucaagcua

**Figure 5:**
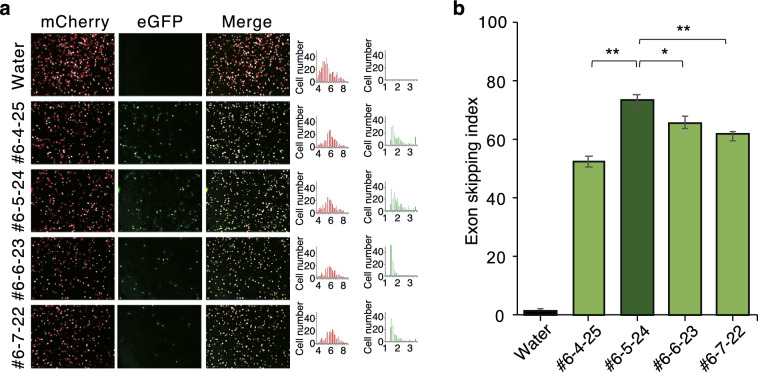
2′-OMe RNA-ASO screening. (**a**) HeLa cells were transfected with FMv2WDR45_int5m and ASO WDR45In5#6-4-25, #6-5-24, #6-6-23, #6-7-22, and their respective skipping effects were examined after 24 h. The respective images of mCherry and eGFP and superimposed images are shown. Also shown is a histogram of the distribution of luminance of mCherry and eGFP. (**b**) Skipping index of pseudoexons is shown; the effect of ASO WDR45In5 #6-5-24 was significantly higher than that of other ASOs; P* < 0.05.

HeLa cells were transfected with FMv2WDR45_int5mut and ASO at a final concentration of 100 nM, and fluorescence was observed by fluorescence microscopy after 24 h. The results showed that ASOs with sequences complementary to the 3′ end region of the pseudoexon and the c.235 + 159C > G mutation were highly active in inducing pseudoexon skipping. Therefore, we narrowed the target sequence of ASOs to the splice donor site of the pseudoexon and synthesized and evaluated a total of 21 additional ASOs with difference length (Fig. 4, Table 2). The screening results of the four ASOs in the final stage of the evaluation are shown (Green bars in Fig. 4). The results showed that the 24-base ASO, WDR45In5#6-5-24 (Fig. 5): caaccuucucaagcuauaagaucc, was the strongest inducer of pseudoexon skipping, and this sequence was selected as a candidate for further development.

**Table 2:** 2′-OMe-ASOs targeting the 3′ end of the pseudoexon.

ASO name	Sequence
WDR45In5#6-3-30;	accaaccuucucaagcuauaagauccauga
WDR45In5#6-3-27;	accaaccuucucaagcuauaagaucca
WDR45In5#6-3-26;	accaaccuucucaagcuauaagaucc
WDR45In5#6-3-25;	accaaccuucucaagcuauaagauc
WDR45In5#6-3-24;	accaaccuucucaagcuauaagau
WDR45In5#6-3-21;	accaaccuucucaagcuauaa
WDR45In5#6-4-26;	ccaaccuucucaagcuauaagaucca
WDR45In5#6-4-25;	ccaaccuucucaagcuauaagaucc
WDR45In5#6-4-24;	ccaaccuucucaagcuauaagauc
WDR45In5#6-4-23;	ccaaccuucucaagcuauaagau
WDR45In5#6-5-25;	caaccuucucaagcuauaagaucca
WDR45In5#6-5-24;	caaccuucucaagcuauaagaucc
WDR45In5#6-5-23;	caaccuucucaagcuauaagauc
WDR45In5#6-5-22;	caaccuucucaagcuauaagau
WDR45In5#6-5-21;	caaccuucucaagcuauaaga
WDR45In5#6-5-20;	caaccuucucaagcuauaag
WDR45In5#6-5-19;	caaccuucucaagcuauaa
WDR45In5#6-5-18;	caaccuucucaagcuaua
WDR45In5#6-6-24;	aaccuucucaagcuauaagaucca
WDR45In5#6-6-23;	aaccuucucaagcuauaagaucc
WDR45In5#6-7-22;	accuucucaagcuauaagaucc

### Optimization of antisense oligonucleotides

2′-O-methyl RNA was used to clarify the optimal sequence for exon skipping induction. Thereafter, ENA (2′-O,4′-C-ethylene bridged nucleic acid) (ENA; registered trademark of Daiichi Sankyo Company, Limited, Tokyo, Japan)^12^, a modified nucleic acid expected to exon skipping induction strongly, was used to provide nuclease resistance and high affinity for the complementary sequence (Table 3). Based on the structural characteristics of previous cases in which ENA-modified nucleic acids have been used in clinical trials^13^, two ENA-ASOs (ENAa and ENAb) were synthesized and their activities were evaluated.

**Table 3:**
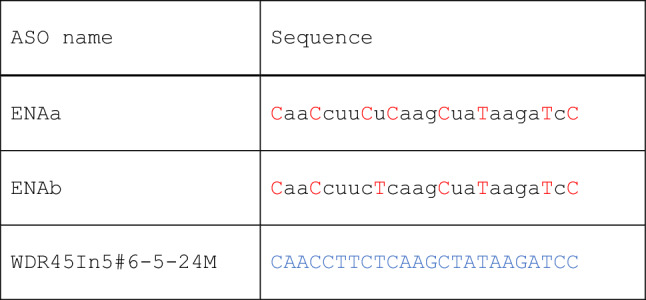
Modified ASOs.

Blue letters in uppercase: PMO.

Red letters in uppercase: ENA.

Black letters in lowercase: 2′-OMe RNA.

**Figure 6:**
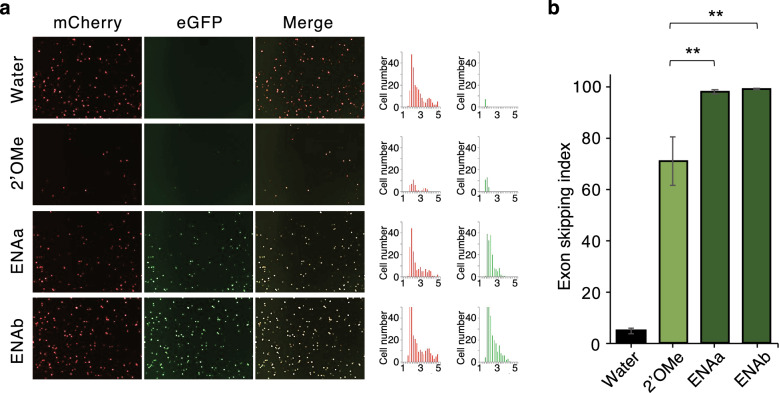
ENA-ASO evaluation in HeLa Cells. (**a**) HeLa cells were transfected with FMv2WDR45_int5 m and three ASOs, and their respective skipping was evaluated by fluorescence microscopy after 24 h. (**b**) The skipping index is shown graphically; both ENA-ASOa and ENAb showed significantly higher exon skipping index than 2′OMe, P** < 0.01.

HeLa cells were transfected with FMv2WDR45_ int5mut and ASO at a final concentration of 100 nM, and fluorescence was observed by fluorescence microscopy after 24 h. The results showed that both two types of ENA-ASO exhibited higher activity than 2′-OMe-RNA (Fig. 6).

**Figure 7:**
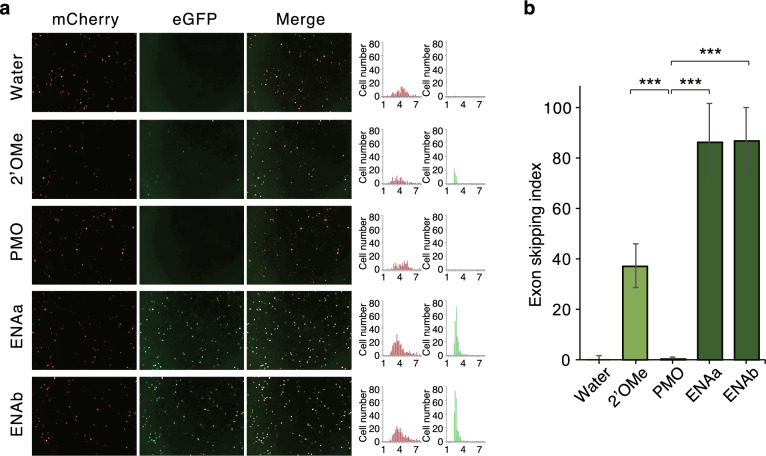
ENA-ASO evaluation in SH-SY5Y Cells. (**a**) SH-SY5Y cells were transfected with FMv2WDR45_int5m and ASO and their respective skipping effects were examined 24 h later. (**b**) Skipping indices are shown in the graph: 2′OMe (2′OMe RNA-ASO); WDR45In5#6-5-24, PMO (morpholino-ASO); WDR45In5#6-5-24 M, ENAa; WDR45In5#6-5-24Ea, ENAb; WDR45In5#6-5-24Eb. P*** < 0.0001.

Since the therapeutic target in BPAN is brain cells, we investigated whether this ENA-ASO is effective in neuronal cells in vitro. Cultured SH-SY5Y cells were simultaneously transfected with the construct and ASO at a final concentration of 100 nM and evaluated by fluorescence microscopy^14^. The results showed that the skipping effect of ENA-ASO was high in neuronal cells, indicating that these are effective (Fig. 7). In order to compare ENA-ASO with morpholino nucleic acids (WDR45In5#6-5-24 M) (Table 3), which are currently widely utilized as antisense oligonucleotides, we synthesized ASO with morpholino nucleic acids from the previous nucleotide sequence and evaluated the skipping of pseudoexons. The results showed that ENA-ASO has a stronger ability to induce exon skipping than morpholino nucleic acid (Fig. 7). Therefore, we conclude that ENA-ASO is the best antisense oligonucleotide for application to patients.

### Addition of antisense oligonucleotides to the mutant vector system with exons

The optimized exon-suppressing ASO (WDR45In5#6-5-24, ENAb) was added to the mutant vector system with exons 3 to 7 (pcWDR45Ex3-7mut) with the c.235 + 159C > G variant.

We performed Nanopore long read sequencing of the RT-PCR products amplified from spliced transcripts spanning from exon 3 to exon 7. As shown in Supplementary Fig. 3, expression of a transcript with a normal splicing pattern spanning from exon 3 to exon 7 and devoid of pseudo-exon(s) was observed only when the optimized exon-suppressing ASO (WDR45In5#6-5-24, ENAb) was added to the mutant vector system with exons 3 to 7 (pcWDR45Ex3-7mut) with the c.235 + 159C > G variant.

## Discussion

Here we reported a patient with BPAN who had a heterozygous intronic pathogenic *WDR45* variant which cause abnormal splicing: inclusion of a 100 bp-long pseudoexon or less abundant 127 bp long pseudoexon. The result can be interpreted as follows: the intronic variant results in the generation of an aberrant transcript because the creation of novel splicing donor site and use of cryptic splicing acceptor sites. The abnormal spicing patterns exon 3–exon 4–pseudoexon–exon 6–exon 7 or exon 3–exon 4–pseudoexon–exon 7 were successfully recapitulated in an minigene assay.

The mutual exclusion of exon 5 and φ100/φ127 would be explained by alternative splicing theories involving exons with "weak" splice sites. Although the splicing scores of wild-type exon 5 are stronger than those of φ100/φ127, exon 5 has relatively "weak" splice sites because exon 5 can be excluded from transcripts in wild-type cells^15^. When two exons with relatively weak splice sites are in close proximity and the size of the intervening intron is less than 60 base pairs, mutually exclusive alternative splicing occurs through a mechanism called steric hindrance^16^. Since the distance between the 3′ end of exon 5 and φ100 was 58 bp and that between the 3′ end of exon 5 and the 5′ end of φ127 was 31 bp, which is less than 60 bp, steric hindrance can explain the observation that exon 5 and φ100/φ127 are included in the spliced transcript in a mutually exclusive manner.

Present study illustrates experimental utility of dual fluorescence splicing reporter minigene system in high-throughput screening therapeutically effective antisense oligonucleotides and will be readily applicable to any patients with splicing abnormalities of various genes^11^.

It is interesting to note that both in the in vivo transcriptome data from peripheral blood and in vitro minigene assay showed that exon 5 tended to be excluded from the abnormal transcript which included the pseudoexon. In other words, the psuedoexon and exon 5 were included in a mutually exclusive manner. Exclusion of exon 5 in the alternatively spliced aberrant transcript may be causally related with the observation that alternative isoform devoid of exon 5 is relatively abundant in peripheral blood from a normal individual and also abundant in RT-PCR product from the minigene. Indeed, various computer prediction programs showed that the strength of non-canonical donor site is as strong as those of the canonical donor site of intron 5.

In our study, the results of a hybrid minigene assay containing the patient's pathogenic intronic variant were comparable to those of transcript analysis using the patient's peripheral blood. Based on this agreement, we expect that an antisense oligonucleotide that suppresses abnormal pseudoexon formation (i.e., WDR45In5#6-5-24) will be an excellent candidate that will be therapeutically effective in vivo if further studies show that the exon-suppressing antisense oligonucleotide can indeed correct splicing in patient-derived living cells such as cultured fibroblasts.

We tried to find antisense oligonucleotides that could suppress formation of pseudoexon using ASO with various chemical modifications. Initial screening was accomplished through 2′-OMe-RNA. Based on the results of the initial screening, four ENA-modified nucleic acids were tested because of their clinical utility despite high cost. It is interesting to note that pseudoexon-skipping efficiency of various ASOs was tissue dependent: Efficiency of both 2′-OMe-RNA and ENA-modified nucleic acids were both lower in neuronal cells than in HeLa cells. Reduction in efficiency was less pronounced when ENA-modified nucleic acids were used (Figs. 6 and 7). Surprisingly enough, morpholinos which have been used for clinical purposes (Fig. 7), had very low efficiency in inducing of skipping of pseudoexon. Overall, these data collectively showed promising therapeutic potential of ENA-modified ASOs.

A follow-up experiment with the exon-suppressing ASO (WDR45In5#6-5-24, ENAb), showed that the addition of the ASO to the mutant vector system using HeLa cells with exons 4 to 7 (pcWDR45Ex3-7mut) led to expression of a transcript with a normal splicing pattern spanning from exon 3 to exon 7 and devoid of pseudo-exon(s) (Supplementary Fig. 3).

In conclusion, this study provides proof of splicing abnormalities associated with variant in *WDR45*. Among neurodevelopmental disorders with congenital onset, natural course of BPAN is unique in that devastating extrapyramidal manifestations start in the second decade of life in addition to developmental delay that is apparent immediately after birth. Hence, prevention or attenuation of the second phase (i.e., extrapyramidal degeneration) through oligonucleotide therapy would have significant clinical value.

## Materials and methods

### Ethical adherence and conflicts of interest

The study was approved by the central ethics committee of central institutional review board at Tohoku University in Japan (approval number: 20851). The methods were carried out in accordance with the approved guidelines and the Declaration of Helsinki. Written informed consent from the parents and approval from the ethic committee of local institutional review board were obtained for the molecular studies. Thereafter, the patient was recruited through the “Initiative on Rare and Undiagnosed Disease” project^17^ and peripheral blood samples were obtained from the patient and her parents. There was no conflict of interest.

### Genomic analysis

Genomic DNA was extracted from the peripheral blood leukocytes of the patient and her parents. An exome analysis was performed as previously reported^18^. Briefly, mapping of the sequence reads to the human reference genome (GRCh37) was performed according to the Burrows-Wheeler Aligner (BWA)^19^ and the Genome Analysis Tool Kit (GATK)^20^ best-practice guidelines, as packaged in the integrated analysis suite variant tools^21^. The called variants were annotated with SnpEff^22^. SpliceAI and PDIVAS as used to predicted effects of variants in splicing. PDIVAS prediction score was obtained from the pre-computed score file distributed by developers^7,8^.

Whole-genome sequencing was performed as follows: genomic DNA from the blood was prepared using the TruSeq PCR-Free HT library prep kit (Illumina) after mechanical shearing using a Covaris ultrasonicators. Libraries were sequenced to 129 giga bases in 2 × 150 paired reads on a NovaSeq6000. The raw reads were mapped to reference sequence GRCh37 and variants were called using DRAGEN 3.5 (Illumina).

ESEfinder^23^ was used to search for putative exonic splicing enhancers (ESE) using matrices for four Ser/Arg-rich proteins (SR proteins), a family of conserved splicing factors including SC35 (SRSF2). For SRSF2, a score is considered high if it is greater than 2.383.

Transcriptome analysis was performed using total RNA extracted from peripheral blood. The Globin-Zero Gold rRNA Removal Kit (Illumina) was used to remove rRNA according to the manufacturer's protocol. Paired-end RNA-Seq libraries were prepared using the Illumina TruSeq Standard mRNA Sample Prep kit (Illumina). All the samples were sequenced on an Illumina HiSeq 2500 platform (100-nucleotide read length). In total, we obtained 147 million paired end reads per sample. An analysis method for detecting abnormally expressed genes, known as OUTRIDER, was used to examine the proposita's RNA sequencing data^24^.

### Insertion of exon 5 for analysis of exonization of intron sequences

We inserted the sequence around exon 5 of WDR45 into this H492 and analyzed the splicing products to prove pseudoexon formation in vitro. The genomic region of normal individuals of various sizes around exon 5 was PCR amplified and the product was inserted into H492. In addition, a PCR mutagenesis method was used to create a single nucleotide substitution in the normal genome to construct a modified H492 with the same abnormal sequence as the patient. The constructed modified H492 was then transfected and expressed in HeLa cells, and the expression product was analyzed by RT-PCR.

### Insertion of exons 3 to 7 for analysis of exonization of intron sequences

We insert a 1.7 kb region of exons 3 to 7 directly into pcDNA3.1( +) and a set of primers (forward primer WDR45Ex3F: 5′-GTTTAAACTTAAGCTAGGAACAATCCTGCACCA-3′ and reverse primer WDR45Ex7R: 5′-CTGGACTAGTGGATCCCTTGGGGTTGTCCCGGG-3′, respectively, homologous sequences to the vector for In-fusion cloning are underlined) was used to amplify from the genomic region of normal individuals. PCR products were cloned into pcDNA3.1(+) pre-digested with the respective enzymes (HindIII-HF: R3104S, BamHI: R0136S, New England Biolabs, Ipswich, MA, USA) and designated pcWDR45Ex3-7. After confirming this construct by sequencing, the construct was subjected for PCR amplification using a primer set (WDR45c235 + 159CGF: 5′-TGAGAAGGTTGGTGCTTTGTTTTCAT-3′, WDR45c235 + 159CGR: 5′-GCACCAACCTTCTCAAGCTATAAGAT-3′) to introduce the nucleotide change. The resulting constructs were sequenced and designated pcWDR45Ex3-7mut. They were introduced into HeLa cells and the products were subjected to RT-PCR analysis.

The method was as follows: 1 mL of DMEM (Nacalai Tesque, Kyoto, Japan) containing 200,000 HeLa cells (American Tissue Culture Collection, Manassas, VA, USA) was added to one well of a 12-well plate. The culture medium was then incubated with 500 ng of pcWDR45Ex3-7 or pcWDR45Ex3-7mut premixed with Lipofectamine 3000 Transfection Reagent (L3000015, Invitrogen, Thermo-Fisher Scientific, Waltham, MA, USA) 4 μL, P3000 (L3000015, Invitrogen, Thermo-Fisher Scientific, Waltham, MA, USA) 1 μL, Opti-MEM 200 μL at room temperature for 15 min. Then 800 μL of DMEM medium was added to the mixture. After another 24 h of incubation, the cells were subjected to mRNA analysis. Total RNA was extracted using the RNeasy Plus Mini Kit (Qiagen), and RT-PCR was subsequently performed. cDNA was synthesized from 0.5 μg of each RNA using random primers (48190011, Thermo Fisher Scientific) and M-MLV Reverse Transcriptase (28025021, Thermo Fisher Scientific). Transcripts were PCR amplified using a set of primers (T7-F; 5′-TAATACGACTCACTATAGGG-3′ and BGH-R; 5′-TAGAAGGCACAGTCGAGG-3′). PCR amplification was performed using 2 µL of cDNA, 2 µL of 10 × ExTaq buffer, 0.25 U of ExTaq polymerase (RR001B, Takara Bio), 500 nM of each primer, and 200 µM dNTPs in a total volume of 20 µL. For amplification of transcripts, 30 cycles of amplification were performed on a Mastercycler Gradient PCR (Eppendorf) under the following conditions: initial denaturation at 94 °C for 3 min, followed by denaturation at 94 °C for 0.5 min, annealing at 60 °C for 0.5 min and extension at 72 °C for 1.5 min. For GAPDH amplification, 18 cycles of amplification were performed. PCR amplified products were electrophoresed using DNA 1000 LabChip kits on an Agilent 2100 Bioanalyzer (Agilent Technologies, Santa Clara, CA, United States), and the separated band was semi-quantified by its density. The same assay was performed with pcWDR45Ex3-7mut and ENAa or ENAb (see Results section) in the HeLa cell system described above.

To analyze the full length of transcripts from the vectors, Nanopore long-read sequencing were performed. Nanopore sequencing libraries were prepared using the above RT-PCR products as templates. The sequencing libraries were prepared with Ligation Sequencing Kit V14 (Oxford Nanopre Technologies, UK) and sequenced with P2 Solo (Oxford Nanopre Technologies) using a PromethION flowcells (R10.4.1, Oxford Nanopre Technologies). The bases calling of sequencing reads were performed using Dorado (v.0.4.3), and the sequencing reads were mapped to human reference genome (hg38) using minimap2 (v.2.24-r1122) with "-x splice" option.

### Construction of a mini-gene for detecting appropriate antisense olionucleotides and evaluation by fluorescence.

A fragment of intron 5 of WDR45 was PCR amplified using PrimeStar Max (Takara) and a set of primers (forward primer WDR45In5PseudoF: 5′-TGCTTTGTTTAAAGCTTGCCCTCATCCTGCCCTTTGG-3′ and reverse primer WDR45In5PseudoR: 5′-CTGGGACTTAGTGGATCCCAGGGACCTCAACCTACTT-3′, respectively, homologous sequences to the vector for In-fusion cloning are underlined) was used to amplify from the genomic region of normal individuals; PCR products were introduced into FMv2 pre-digested with restriction enzymes (HindIII-HF, BamHI). The resulting construct was used as a template to introduce a single nucleotide substitution of c.235 + 159C > G by mutagenesis PCR method.

These constructs were transfected into HeLa cells and observed for fluorescence. Red signals are those generated in cells that have successfully transfected the mini-gene and express the mCherry protein. The green signal is generated by successful transfection of the mini-gene and translation of the mature eGFP mRNA after splicing out the intervening segment; when the two images are combined, a yellow signal is detected, indicating co-expression of the red and green signals. To quantify cells positive for red and green, a histogram representing fluorescence intensity was created and the number of cells was calculated from the histogram. Fluorescence intensity (*F*_*I*_) was calculated using the following equation:

$$F_{I} = \left( {{\text{fluorescence}}} \right)/\left( {{\text{area of each cell }}\left( {\mu {\text{m}}^{{2}} } \right)} \right)$$

Exon skipping index (*E*_*si*_) was calculated as follows:

$$E_{si} = {1}00*G_{n} /R_{n}$$

*G*_*n*_, number of green eGFP-positive cells; *R*_*n*_, number of red mCherry-positive cells. The average was obtained from three independent experiments.

HeLa cells (American Tissue Culture Collection, Manassas, VA, USA) were cultured in 0.1 mL of DMEM (Nacalai Tesque, Kyoto, Japan) containing 20,000 cells per well of a 96-well plate. After 24 h, the medium was replaced with 80 μL of FluoroBrite DMEM medium (A1896701, Gibco by Thermo-Fisher Scientific, Waltham, MA, USA) and 50 ng of FMv2, FMv2 WDR45_int5 or FMv2WDR45_int5mut premixed Lipofectamine 3000 Transfection Reagent (L3000015, Invitrogen, Thermo-Fisher scientific, Waltham, MA, USA) 0.4 μL, P3000 (L3000015, Invitrogen, Thermo-Fisher scientific, Waltham, MA, USA) 0.1 μL, Opti-MEM 20 μL at room temperature for 15 min was added. After 24 h of culture, the cells were subjected to fluorescence microscopy.

### Image analyses

Cells in a microscopic field of each well were imaged using a fluorescence microscope (BZ-X710, Keyence, Osaka, Japan) with a replaced objective (PlanFluor4 × , Nikon Instruments Inc., Tokyo, Japan). Filters were BZ-X GFP (OP-87763, Keyence, Osaka, Japan) for GFP detection and BZ-X TexasRed (OP-87765, Keyence, Osaka, Japan) for mCherry detection. Images were optimized using BZ-Viewer (Keyence, Osaka, Japan). Cell counting and quantification of fluorescence intensity were performed using Hybrid Cell Count (BZ-H3M, Keyence, Osaka, Japan). Fluorescence intensity was determined using the following formula: fluorescence strength (FS) = fluorescence/area of each cell (µm2). Exon skipping index was calculated as follows: (the number of green GFP-positive cells)/(the number of red mCherry-positive cells) × 100.

### Statistical analyses

Results were expressed as mean + / − standard deviation (SD) and compared using Student's t-test for comparisons between two groups. All statistical analyses were performed using SPSS software (version 17.0; SPSS, Inc. Chicago, IL, United States), and *P* < 0.05 was considered statistically significant.

### Supplementary Information

Supplementary Information.

## Data Availability

The ClinVar accession numbers for the *WDR45* variant reported in this paper is SCV004032199 (ClinVar, https://www.ncbi.nlm.nih.gov/clinvar/).
