# Supplementary material for: Successful skipping of abnormal pseudoexon by antisense oligonucleotides in vitro for a patient with beta-propeller protein-associated neurodegeneration
Source: Sci Rep. 2024 Mar 18;14:6506. doi: 10.1038/s41598-024-56704-z (PMC10948761; doi:10.1038/s41598-024-56704-z)
Supplement: Supplementary file 1 — Supplementary Information. [file 41598_2024_56704_MOESM1_ESM.pdf]

**a**

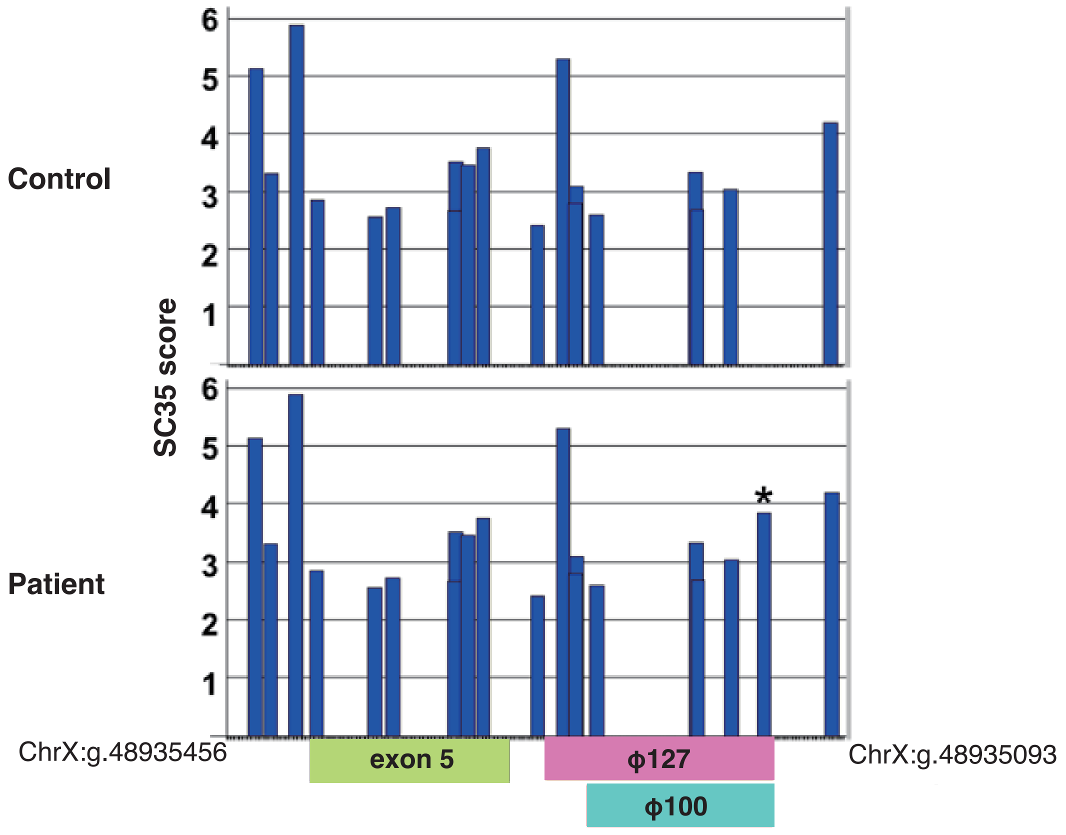


**b**

```
                                                                              exon 5
WT       tgggctgggcattggctcccacctccactgacaccctggtccctgtccagACCACGAGCAGGTGGGCAGCATGGGCTTGGTGG
Pt φ100  tgggctgggcattggctcccacctccactgacaccctggtccctgtccagaccacgagcaggtgggcagcatgggcttggtgg
Pt φ127  tgggctgggcattggctcccacctccactgacaccctggtccctgtccagaccacgagcaggtgggcagcatgggcttggtgg

WT       AGATGCTGCACCGCTCCAACCTTCTGGCCTTGGTGGGCGGTGGTAGTAGTCCCAAGTTCTCAGAGATCTCAGgtaagtgccct
Pt φ100  agatgctgcaccgctccaaccttctggccttggtgggcggtggtagtagtcccaagttctcagagatctcaggtaagtgccct
Pt φ127  agatgctgcaccgctccaaccttctggccttggtgggcggtggtagtagtcccaagttctcagagatctcaggtaagtgccct

                               ϕ127                              ϕ100
WT       catcctgccctttggcccagatttctcggattcctggcctcccacaggcaccccaaggtactggcagatgaagacgtcagagt
Pt φ100  catcctgccctttggcccagatttctcggattcctggcctcccacagGCACCCCAAGGTACTGGCAGATGAAGACGTCAGAGT
Pt φ127  catcctgccctttggcccagATTTCTCGGATTCCTGGCCTCCCACAGGCACCCCAAGGTACTGGCAGATGAAGACGTCAGAGT

WT       acttcagagtcacacagagaggaggcctacagcttggaagtcatggatcttatagcttgagaagcttggtgcttttgttttca
Pt φ100  ACTTCAGAGTCACACAGAGAGGAGGCCTACAGCTTGGAAGTCATGGATCTTATAGCTTGAGAAGgttggtgcttttgttttca
Pt φ127  ACTTCAGAGTCACACAGAGAGGAGGCCTACAGCTTGGAAGTCATGGATCTTATAGCTTGAGAAGgttggtgcttttgttttca
```

**Supplementary Figure 1.** putative exonic splicing enchancers (ESEs). (**a**) ESE-Finder predicted that the intronic variant c.235+159G>C would create a potential SRSF2 (SC35) binding site with a score of 3.84, which was higher than the threshold of 2.383. The distribution of putative ESEs (i.e., scores higher than 2.383) surrounding exon 5, ϕ127, ϕ100 and corresponding reference sequences (i.e., wild type) are shown. (**b**) Detailed information on the nucleotide sequences of the reference and mutant sequences around exon 5, ϕ100, and ϕ127 in conjunction with the putative ESEs. Underlined bases indicate ESE. The yellow-green region indicates exon5, the cyan region indicates ϕ100, and the magenta region indicates ϕ127. Base in red letter indicates mutation observed in the patient. Bolded “gt” and “ag” indicate introns at the splice site.

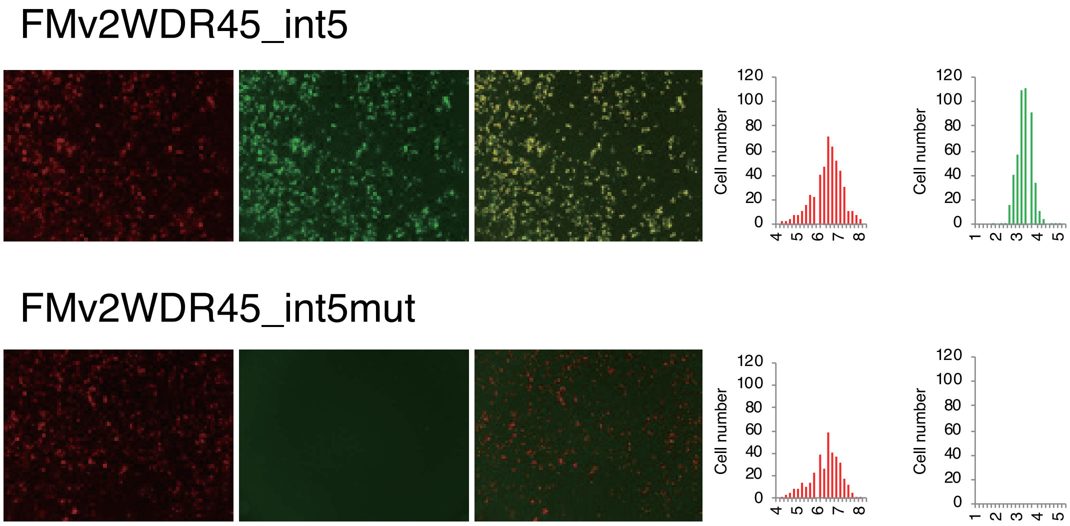


**Supplementary Figure 2.** Skipping effects of FMv2WDR45_int5 or FMv2WDR45_int5mut in HeLa cells. HeLa cells were transfected with FMv2WDR45_int5 or FMv2WDR45_int5mut, and their respective skipping effects were examined after 24 h. The respective images of mCherry and eGFP and superimposed images are shown. Also shown is a histogram of the distribution of luminance of mCherry and eGFP.

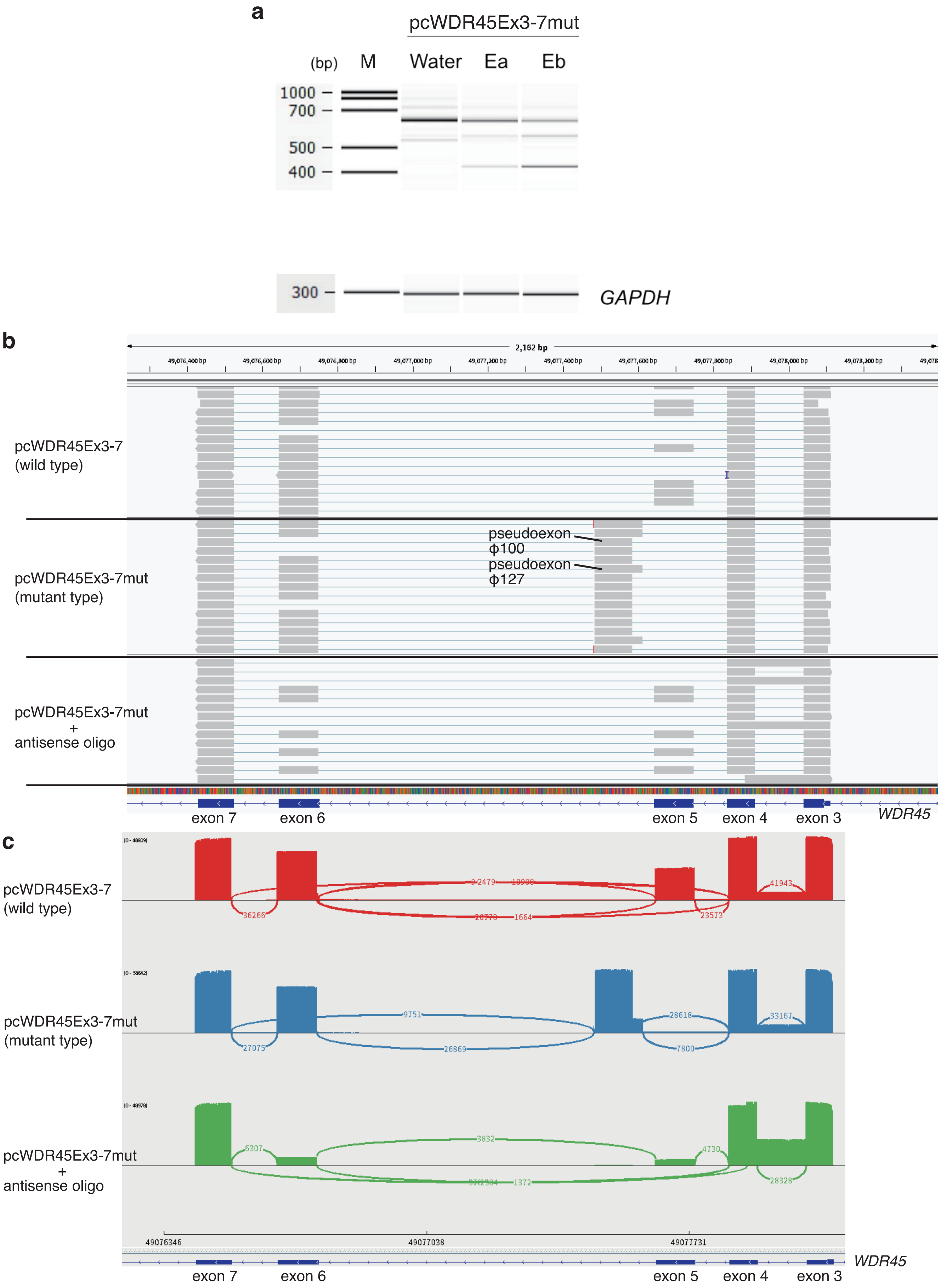


**Supplementary Figure 3.** Transcriptional changes due to mutation and and addition of antisense oligos. (**a**) Suppression of pseudoexons by ENAb in the minigene pcWDR45Ex3-7mut. The minigene pcWDR45Ex3-7mut and ENAa or ENAb were introduced into HeLa cells and the mRNA was analyzed by RT-PCR after 24 hours of antisense oligonucleotide. Splicing products of the minigene were shown. Complete images of the gel electrophoresis were shown in Supplementary Fig. 4c.M: molecular weight markers, Water: negative control, Ea: ENAa as antisense oligonucleotide, Eb: ENAb as antisense oligonucleotide. (**b**) Results of Nanopore long sequencing of RT-PCR products. From top to bottom, the results are shown for the wild type pcWDR45Ex3-7 plasmid, the mutant type pcWDR45Ex3-7mut, and the mutant type pcWDR45Ex3-7mut with antisense oligos (WDR45In5#6-5-24, ENAb). The figures are depicted in the IGV viewer. (**c**) Sashimi plot of sequencing reads of the RT-PCR products above.

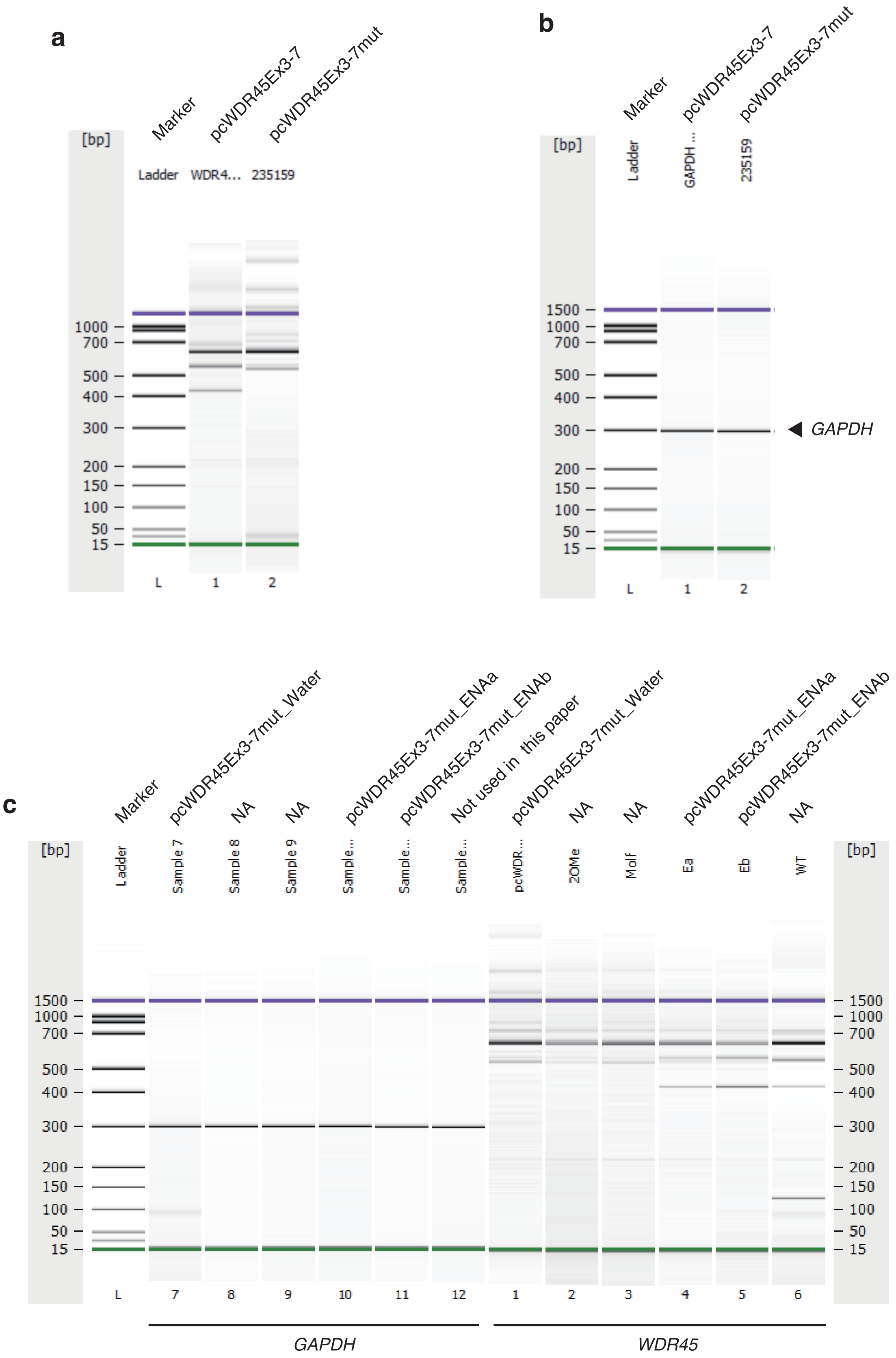


**Supplementary Figure 4.** Original complete electrophoresis gel images of Figure 3 and Supplementary Figure 3.(a) RT-PCR results for WDR45 in Figure 3. (b) RT-PCR results for GAPDH in Figure 3. (c) The left half shows the RT-PCR results for GAPDH and the right half for WDR45 in Supplementary Figure 3. NA, not available (i.e., not used) in this paper.

**Supplementary Table 1.** Computer prediction programs predicted that the non-canonical splicing acceptor sites

| | SpliceSiteFinder | MaxEnt | NNSPLICE | GeneSplicer |
|---|---|---|---|---|
| Range | [0-100] | [0-16] | [0-1] | [0-21] |
| Threshold values for splicing acceptor sites | ≧70 | ≧0 | ≧0.4 | ≧0 |
| intron 5– c.235+32 | 76.22 | 6.55 | 0.68 | 5.07 |
| intron 5– c.235+59 | 81.84 | 6.75 | 0.92 | 6.74 |
| Splice acceptor site of exon 4 | 100 | 10.86 | 1.00 | 11.04 |
